# Multi-View Structural Local Subspace Tracking

**DOI:** 10.3390/s17040666

**Published:** 2017-03-23

**Authors:** Jie Guo, Tingfa Xu, Guokai Shi, Zhitao Rao, Xiangmin Li

**Affiliations:** 1 Image Engineering&Video Technology Lab, School of Optoelectronics, Beijing Institute of Technology, Beijing 100081, China; jieguo_2013@163.com (J.G.); shi_guokai_123@126.com (G.S.); rzt115126@163.com (Z.R.); li_xiangmin@bit.edu.cn (X.L.); 2 Key Laboratory of Photoelectronic Imaging Technology and System, Ministry of Education of China, Beijing 100081, China

**Keywords:** visual tracking, sparse representation, structural local appearance model, multi-view, PCA

## Abstract

In this paper, we propose a multi-view structural local subspace tracking algorithm based on sparse representation. We approximate the optimal state from three views: (1) the template view; (2) the PCA (principal component analysis) basis view; and (3) the target candidate view. Then we propose a unified objective function to integrate these three view problems together. The proposed model not only exploits the intrinsic relationship among target candidates and their local patches, but also takes advantages of both sparse representation and incremental subspace learning. The optimization problem can be well solved by the customized APG (accelerated proximal gradient) methods together with an iteration manner. Then, we propose an alignment-weighting average method to obtain the optimal state of the target. Furthermore, an occlusion detection strategy is proposed to accurately update the model. Both qualitative and quantitative evaluations demonstrate that our tracker outperforms the state-of-the-art trackers in a wide range of tracking scenarios.

## 1. Introduction

Visual tracking plays an important role in computer vision and has received fast-growing attention in recent years due to its wide practical application. In generic tracking, the task is to track an unknown target (only a bounding box defining the object of interest in a single frame is given) in an unknown video stream. This problem is especially challenging due to the limited set of training samples and the numerous appearance changes, e.g., rotations, scale changes, occlusions, and deformations.

To solve the problem, many effective trackers have been proposed [1,2,3,4] in recent years. Most methods are developed from the discriminative or generative perspectives. Discriminative approaches use an online updated classifier or regression model to distinguish the object from the background. Avidan [5] uses AdaBoost to combine a set of weak classifiers into a strong classifier to label each pixel and develops an ensemble tracking method. Grabner et al. [6] propose a semi-supervised online boosting algorithm to handle the drift problem in tracking by the usage of a given prior. Babenko et al. [7,8] introduce multiple instance learning (MIL) into online object tracking where bag labels are adopted to select effective features. Hare et al. [9] propose the Struck tracker which directly estimates the object transformation between frames, thus avoiding the heuristic labels of samples. Kalal et al. [10] propose a P-N learning algorithm which uses two experts to estimate and correct the errors made by the classifier and tracker. More recently, Li et al. [11] proposed a novel tracking framework with adaptive features and constrained labels to handle illumination variation, occlusion and appearance changes caused by the variation of positions. Among all of the discriminative approaches, recently, correlation filter-based tracking algorithms [12] have drawn increasing attention because of their dense sampling property and fast computation in the Fourier domain. Bolme et al. [13] propose the MOSSE tracker which finds a filter by minimizing the sum of the squared error between the actual convolution outputs and the desired convolution outputs. The MOSSE tracker can handle several hundreds of frames per second because of the fast element-wise multiplication and division in the Fourier domain. Henriques et al. [14] extend correlation filters to a kernel space, leading to the CSK tracker which achieves competitive performance and efficiency. To further improve the performance, the KCF method [15] integrates multiple features into the CSK tracking algorithm. More recently, Xu et al. [16] proposed a new real-time robust scheme based on KFC to significantly improve tracking performance on motion blur and fast motion.

In contrast, generative methods typically learn a model to represent target object appearances. The object model is often updated online to adapt to appearance changes. Comaniciu et al. [17] use a spatial mask with an isotropic kernel to regularize the histogram-based target representations. The FragTrack [18] represents template objects by multiple image fragments, which addresses the partial occlusion problem effectively. Ross et al. [19] propose the IVT tracker, which incrementally learns a low-dimensional subspace representation of target appearances to account for numerous appearance changes. Sanna et al. [20] propose a novel ego-motion compensation technique for UAVs (unmanned aerial vehicles) which uses the data received from the autopilot to predict the motion of the platform, thus allowing to identify a smaller region of the image (subframe) where the candidate target has to be searched for in the next frame of the sequence. Kwon et al. [21] decompose the observation model into multiple basic observation models to cover a wide range of appearance changes for visual tracking. Lamberti et al. [22] exploit a motion prediction metric to identify the occurrence of false alarms and to control the activation of a template matching (TM)-based phase, thus, improving the robustness of the tracker. 

Among all of the generative approaches, recently, sparse representation-based tracking methods [23] have been developed for object tracking because of their demonstrated good performance in tracking. These methods can be categorized into methods based on a global sparse appearance model [24,25,26,27,28], local sparse appearance model [29,30], and joint sparse appearance model [31,32,33]. The global model represents each target candidate as a sparse linear combination of target templates. These methods can deal with slight occlusions but are less effective in handling heavy occlusions because of the global representation scheme, which loses partial information. Liu et al. [29] proposed a local sparse model with mean-shift algorithm for tracking. However, it is based on a static local sparse dictionary and this is less effective in dealing with severe appearance changes. Jia et al. [30] developed a tracking method based on a structural local sparse appearance model. The representation exploits both partial information and spatial information of the target based on a novel alignment-pooling method. However, it fails to consider the relationship among different candidates and their patches. The joint sparse appearance models [31] aims to exploit the intrinsic relationship among different candidates. The assumption is that the corresponding features of the particles are likely to be similar because of the sample strategy in particle filter-based methods. Then all of the candidates can be jointly represented by the same few target templates. However, when abrupt motion occurs, most candidates will likely be background. In this situation, if the joint sparsity strategy is adopted, the handful of target candidates will be dominated by a big quantity of background candidates, thus failing to represent the target well and causing tracking failure. Zhang et al. [33] proposed a structural sparse tracking algorithm which combines global and partial models together, then used the multi-task framework to exploit the intrinsic relationship among different candidates and their local patches. However, it also cannot well represent the target object when abrupt motion occurs. Zhuang et al. [27] proposed a multi-task reverse sparse representation formulation. In the formulation, they use a Laplacian regularization term to preserve the similarity of sparse codes for the similar candidate features. However, the candidates which have similar features will have similar sparse codes even if the formulation does not contain the Laplacian regularization term. Additionally, all of these methods preserve the information of the target object’s appearances only with a couple of previous time instants, thus, they cannot cover numerous appearances of the target object.

**Figure 1:**
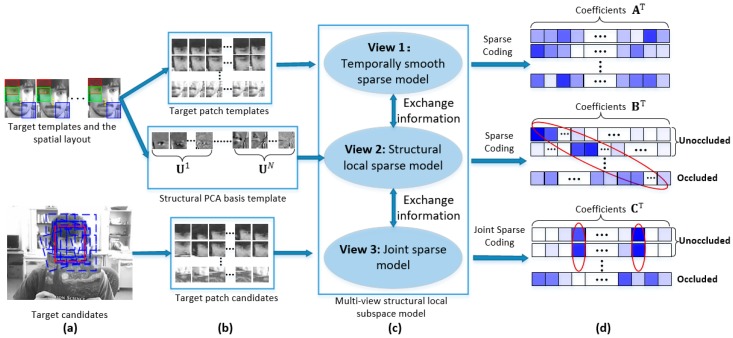
The multi-view structural local subspace model. (**a**) The target templates, the spatial layout, and the target candidates; (**b**) the patch templates, structural PCA basis template, and the patch candidates; (**c**) the three sub-models and the unified model; and (**d**) the sparse coefficients of the three sub-models.

Motivated by the above discussions, we propose a novel multi-view structural local subspace model as shown in Figure 1. For each view, we build a sub-model to exploit the useful information in the view. The whole model iteratively exchanges information among three sub-models. In the target template view, each patch of target object is sparsely represented by the target patch templates independently with a temporally smooth regularization term. The target templates have a strong representation of the current object’s appearance. We use them to account for the short-term memory of target object. In the PCA Eigen template view, we construct a structural local PCA Eigen dictionary to exploit both partial information and spatial information of the target object with sparse constraint. Additionally, the PCA Eigen template model has the ability to effectively learn the temporal correlation of target appearances from past observation data by an incremental SVD update procedure, thus, it can cover a long period of target appearances. We use it to account for the long-term memory of the target. In the target candidate view, we use a Laplacian regularization term to keep the similarity of sparse codes among those unoccluded patches and keep the independence of sparse codes which belong to the occluded patches by an occlusion indicator matrix. Note that the use of the Laplacian regularization term in our model is more meaningful than it is in [27]. The whole model has many good properties. It takes advantages of both sparse representation and incremental subspace learning. This makes the model less sensitive to incorrect updating and makes the model have a proper memory of the target appearances. The model exploits the intrinsic relationship among different target candidates and their local patches, forming a strong identification power to locate the target from many candidates. It can also estimate the reliability of different local patches. This causes the model make full use of the reliable patches and ignore the occluded patches.

We built the model to deal with many tracking problems, e.g., occlusion, deformation, fast motion, illumination variation, scale variation, background clutters, etc. The sparse representation-based tracking method can handle partial occlusion and background clutter to some extent, and the incremental learning of the PCA subspace representation can effectively and efficiently deal with appearance changes caused by rotations, scale changes, illumination variations, and deformations. The proposed tracker takes advantages of both methods, and by considering time consistency, intrinsic relationships among target candidates and their local patches, different reliability of different patches, and the rational update strategy, the proposed method significantly improves the robustness of tracking performance.

The main contributions of this paper are as follows:

(1) A novel multi-view structural local subspace tracking method is proposed. The model jointly takes advantages of three sub-models by a unified objective function which is proposed to integrate the three sub-models together. The proposed model not only exploits the intrinsic relationship among target candidates and their local patches, but also takes advantages of both sparse representation and incremental subspace learning.
(2) We propose an algorithm which can solve the optimization problem well by three customized APG methods, together with an iteration manner.
(3) An alignment-weighting average method is proposed to exploit the complete structure information of the target for robust tracking.
(4) A novel update strategy is developed to account for both short-term memory and long-term memory of target appearances.
(5) Experimental results show that the proposed method outperforms twelve state-of-the-art methods in a wide range of tracking scenarios.

The rest of the paper is organized as follows: In Section 2, we introduce the multi-view structural local subspace model in detail. The optimization of the unified objective function and the overall tracking algorithm are presented in Section 3. Details of the quantitative and qualitative experiments of our method compared with the state-of-the-art methods are discussed in Section 4. In Section 5, we reach the conclusions of the paper.

## 2. Multi-View Structural Local Subspace Model (MSLM)

Most tracking methods use only one clue to model the target appearance. However, only one clue can hardly handle the complicated circumstances that visual tracking faces. Some methods try to fuse different models together to use all of their advantages, but they either simply combine these models or increase the computation burden by using some complicated models. Our method exchanges information among target templates, PCA bases, and candidates in one model to simultaneously use all of the advantages, while keeping computational complexity favorable.

To better illustrate our model, we assume that the optimal state $x^{*}$ in the current frame is already known and the corresponding observation is $y^{*}$. The state $x^{*}={\left[l_{x},l_{y},\theta ,s,r,\phi \right]}^{T}$ includes six affine parameters, where $l_{x},l_{y},\theta ,s,r,\phi$ denote x,y translations, rotation angle, scale, aspect ratio, and skew, respectively. The observation is extracted according to them. We sample a set of overlapped local image patches inside the target region with a spatial layout illustrated in Figure 1. Then we obtain an optimal patch vector $P^{*}=\left[p_{1}^{*},p_{2}^{*},\ldots ,p_{N}^{*}\right]\in {\mathbb{R}}^{d\times N}$, where d is the dimension of the image patch vector, and N is the number of local patches sampled within the target region. Each column in $P^{*}$ is obtained by $\ell_{2}$ normalization on the vectorized local image patches extracted from $y^{*}$. The goal is to mine the most useful information lying in the target patch templates, patch PCA basis, and candidates’ patches to approximate the optimal observation jointly. First, we approximate the optimal patches $P^{*}$ by exploiting the sparsity in the target patch templates. Second, we construct a structured local PCA dictionary to exploit both partial information and spatial information of the target with a sparse constraint. Third, we adopt a Laplacian term to exploit the intrinsic relationship among target candidates and their local patches. Fourth, we propose a unified objective function to integrate these three models and find an iterative manner to effectively exchange information among all of these three models, thus, taking full advantage of all the three subspace sets simultaneously.

### 2.1. View 1: Approximating the Optimal Observation with Target Templates

We collect a set of target templates $T=\left[T_{1},T_{2},\ldots ,T_{n}\right]$, where n is the number of target templates. Then a set of overlapped local patches are sampled inside each target template using the same spatial layout to construct the patch dictionaries $D^{i}=\left[d_{1}^{i},d_{2}^{i},\ldots ,d_{n}^{i}\right]\in {\mathbb{R}}^{d\times n}$, where i=1,…,N. Dictionary $D^{i}$ denotes the dictionary constructed by the $i^{th}$ local image patches of all these n target templates. Each column in $D^{i}$ is obtained by $\ell_{2}$ normalization on the vectorized grayscale image observations extracted from. We assume that the optimal observation $y^{*}$ and its patch vectors $P^{*}$ has already been known. Then the goal is to find the most useful information in target patches templates which can represent the optimal observation as far as possible. Due to the good modelling ability of sparse representation witnessed in [23], we decided to explore the information in target templates which can reflect the current target state with sparsity constraint:

(1) $$\underset{a_{i}}{\min}\frac{1}{2}{\left\|p_{i}^{*}-D^{i}a_{i}\right\|}_{2}^{2}+\lambda_{1}{\left\|a_{i}\right\|}_{1}+\frac{\lambda_{2}}{2}{\left\|a_{i}-a_{i}^{t-1}\right\|}_{2}^{2},\;s.t.\;a_{i}\geq 0,\;i=1,2,\ldots ,N$$

where $p_{i}^{*}$ denotes the $i^{th}$ optimal patch and $a_{i}\in {\mathbb{R}}^{n\times 1}$ is the corresponding sparse code of that patch; $a_{i}^{t-1}$ is the sparse patch code of last frame; $\lambda_{1}$ and $\lambda_{2}$ controls the regularization amount. The last term in Equation (1) is a temporally smooth term which is derived from the observation that target object in neighboring frames are always very similar to each other.

### 2.2. View 2: Approximating the Optimal Observation with Structural Local PCA Basis

To adapt to the target appearance variations caused by illumination change and pose change, the target templates described in last section are updated dynamically. However, these templates are only obtained from the previous couple of time instants. It is a short-term memory of the target appearances. Thus, they cannot cover the numerous appearance variations well. This can be solved by the Eigen template model which has been successfully used in visual tracking scenarios [34]. The Eigen template model has the ability to effectively learn the temporal correlation of target appearances from the past observation data by an incremental SVD update procedure. The incremental visual tracking (IVT) method [19] presents an online update strategy which can efficiently learn and update a low-dimensional PCA subspace representation of the target object. It has been shown that the incremental learning of the PCA subspace representation can effectively and efficiently deal with appearance changes caused by rotations, scale changes, illumination variations, and deformations. However, the holistic PCA appearance model has been demonstrated sensitive to partial occlusion. Since the underlying assumption of PCA is that the error of each pixel is Gaussian distributed with small variances, but when partial occlusion occurs, this assumption no longer holds. Meanwhile, the holistic appearance model does not make full use of partial information and spatial information of the target and, hence, may fail to track when there is occlusion or similar object in the scene.

Motivated by the above observations, we construct a structural local PCA basis dictionary to linearly represent each patch with $\ell_{1}$-norm constraint. The PCA basis dictionary $U=\left[U^{1},U^{2},\ldots ,U^{N}\right]=\left[u_{1},u_{2},\ldots ,u_{\left(m\times N\right)}\right]\in {\mathbb{R}}^{d\times \left(m\times N\right)}$ is concatenated by the PCA basis component of each partial patch, where m is the number of PCA basis of each patch used to construct U and $U^{i}\in {\mathbb{R}}^{d\times m}$ is the eigenvectors corresponding to the $i^{th}$ patch. The dictionary U is redundant for each patch. We can see that each patch will likely be linearly represented by the eigenvectors corresponding to itself and the coefficients of other eigenvectors will be zeros or close to zero. Thus, with the $\ell_{1}$-norm constraint, each local patch will be represented as the linear combination of a few main eigenvectors in U by solving:

(2) $$\underset{b_{i}}{\min}\frac{1}{2}{\left\|p_{i}^{*}-Ub_{i}\right\|}_{2}^{2}+\mu {\left\|b_{i}\right\|}_{1},$$

where μ is the regularization parameter and $b_{i}\in {\mathbb{R}}^{\left(m\times N\right)\times 1}$ is the corresponding sparse code.

### 2.3. View 3: Approximating the Optimal Observation with Target Candidates

The goal of tracking in the Bayesian framework is to find the combination of candidates or the candidate which can best approximate the optimal state. In every frame, we extract a set of target candidates $=\left[z_{1},z_{2},\ldots ,z_{M}\right]$ according to a candidate state set $X=\left[x_{1},x_{2},\ldots ,x_{M}\right]$, where M is the number of target candidates. The sampling strategy of the candidate state set X will be described in detail later. Like the above two model, we sample a set of overlapped local image patches inside each candidate region with the spatial layout forming a candidate patch dictionary $Y^{i}=\left[y_{1}^{i},y_{2}^{i},\ldots ,y_{M}^{i}\right]\in {\mathbb{R}}^{d\times M}$ in the same way as how dictionary $D^{i}$ is constructed, where i=1,…,N. Then we approximate the optimal observation with target candidates by:

(3) $$\underset{C}{\min}\underset{i}{\sum }\frac{1}{2}{\left\|p_{i}^{*}-Y^{i}c_{i}\right\|}_{2}^{2}+\delta_{1}\underset{i}{\sum }{\left\|c_{i}\right\|}_{1}+\frac{\delta_{2}}{2}\underset{ij}{\sum }{\left\|c_{i}-c_{j}\right\|}^{2}W_{ij}\;s.t.\;c_{i}\geq 0,\;i,j=1,2,\ldots ,N,$$

where $\delta_{1}$ and $\delta_{2}$ are regularization parameters, $c_{i}\in {\mathbb{R}}^{M\times 1}$ is the corresponding sparse code and W is an occlusion indicator matrix with $W_{ij}=1-\max\left(o_{i},o_{j}\right)$, where $o_{i}\in \left[0,1\right]$ is the occlusion rate of the $i^{th}$ patch. Details of the occlusion rate are described in Section 3.2.1. The last term in Equation (3) is a Laplacian regularization term inspired by [27]. Different with [27], our model uses this term to exploit the similarity of sparse codes among different spatial layout patches. Note that the number of different spatial layout patches is N. It is actually a small number which does not increase the computation. The occlusion indicator matrix W can indicate if any two different spatial layout patches are both occluded or not. If both are not occluded, the corresponding factor in W will be large to constrain the two sparse codes to have similar values. If any of the two patches is occluded, the corresponding factor in W will be small, thus letting the model avoid the influence of the occluded patches. Similar to [27], we transform the Laplacian term and the optimization problem is reformulated as:

(4) $$\underset{C}{\min}\underset{i}{\sum }\frac{1}{2}{\left\|p_{i}^{*}-Y^{i}c_{i}\right\|}_{2}^{2}+\delta_{1}\underset{i}{\sum }{\left\|c_{i}\right\|}_{1}+\delta_{2}tr\left(CLC^{Τ}\right)\;s.t.\;c_{i}\geq 0,\;i=1,2,\ldots ,N,$$

where L=D−W is the Laplacian matrix, $\;C=\left[c_{1},c_{2},\ldots ,c_{N}\right],$ the degree of $c_{i}$ is defined as $D_{i}=\overset{N}{\underset{j=1}{\sum }}W_{ij}$ and $D=diag\left(D_{1},D_{1},\ldots ,D_{N}\right)$.

### 2.4. Multi-View Structural Local Subspace Model

In the descriptions of above three view models, we assume that the optimal target state $x^{*}$ and its corresponding observation vector $y^{*}$ have already been known. However, in reality, the goal is to find the optimal state in current frame. From above three subsections, we know the optimal state can be approximated from three different views, and every view has its own advantages against others. Thus, we propose a unified objective function to exchange information among different views and jointly exploit all the advantages by:

(5) $$J\left\{A,B,C\right\}=\left(\underset{i}{\sum }\frac{1}{2}{\left\|Ub_{i}-Y^{i}c_{i}\right\|}_{2}^{2}+\delta_{1}\underset{i}{\sum }{\left\|c_{i}\right\|}_{1}+\delta_{2}tr\left(CLC^{Τ}\right)\right)+\mu \underset{i}{\sum }{\left\|b_{i}\right\|}_{1}\;+\gamma \left(\underset{i}{\sum }\frac{1}{2}{\left\|Ub_{i}-D^{i}a_{i}\right\|}_{2}^{2}+\lambda_{1}\underset{i}{\sum }{\left\|a_{i}\right\|}_{1}+\frac{\lambda_{2}}{2}\underset{i}{\sum }{\left\|a_{i}-a_{i}^{t-1}\right\|}_{2}^{2}\right),$$

where $A=\left[a_{1},a_{2},\ldots ,a_{N}\right]$ and $B=\left[b_{1},b_{2},\ldots ,b_{N}\right]$; γ is a constant that balances the importance between the two terms. The estimated coefficients A, B, and C can be achieved by minimizing the objective function (Equation (5)) with non-negativity constraints:

(6) $$\left\{\hat{A},\hat{B},\hat{C}\right\}=\underset{A,B,C}{\mathrm{argmin}}J\left\{A,B,C\right\},\;s.t.\;A\geq 0\;\mathrm{and}\;C\geq 0.$$

However, there exists no close-form solution for the optimization problem with Equation (6). Thus, we develop an iterative manner to solve it.

## 3. Optimization and the Tracking Algorithm

### 3.1. Optimization

In Equation (6), coefficients A,B, and C are all unknown, making the solution of this problem intractable. In this work, we present an iteration method to search the minima of the optimization problem (Equation (6)). Due to the temporal consistency of target object, the coefficient B is initialized by ${\hat{B}}_{t-1}$ which is estimated from last frame. Then coefficients A,B, and C can be achieved by iteratively solve sub-problems (**a**) and (**b**):

(**a**) Fix B, solve A and C: if B is given, Equation (6) can be separated into two sub-problems:

(7) $$\underset{a_{i}}{\min}\frac{1}{2}{\left\|Ub_{i}-D^{i}a_{i}\right\|}_{2}^{2}+\lambda_{1}{\left\|a_{i}\right\|}_{1}+\frac{\lambda_{2}}{2}{\left\|a_{i}-a_{i}^{t-1}\right\|}_{2}^{2},\;s.t.\;a_{i}\geq 0,\;i=1,2,\ldots ,N,$$

and:

(8) $$\underset{C}{\min}\underset{i}{\sum }\frac{1}{2}{\left\|Ub_{i}-Y^{i}c_{i}\right\|}_{2}^{2}+\delta_{1}\underset{i}{\sum }{\left\|c_{i}\right\|}_{1}+\delta_{2}tr\left(CLC^{Τ}\right)\;s.t.\;c_{i}\geq 0,\;i=1,2,\ldots ,N.$$

These two problems both can be effectively and efficiently solved by the accelerated proximal gradient (APG) method [35]. However, there are differences between them. Coefficient A can be obtained by separately solving each $a_{i}$, while coefficient C needs all $c_{i}$ to be solved simultaneously. Details are described below.

Let $1_{a}\in {\mathbb{R}}^{n}$, $1_{c}\in {\mathbb{R}}^{M}$ and $1_{*}\in {\mathbb{R}}^{N}$ represents the column vectors whose entries are all ones. Let ψ(a) denotes the indicator function defined by:

(9) $$\psi \left(a\right)=\{\begin{array}{cc} 0 & a\geq 0\\ +\infty & \mathrm{otherwise} \end{array}.$$

Then Equations (7) and (8) can be optimized alternately as:

(10) $$\underset{a_{i}}{\min}\frac{1}{2}{\left\|Ub_{i}-D^{i}a_{i}\right\|}_{2}^{2}+\lambda_{1}1_{a}^{T}a_{i}+\frac{\lambda_{2}}{2}{\left\|a_{i}-a_{i}^{t-1}\right\|}_{2}^{2}+\psi \left(a_{i}\right),$$

and:

(11) $$\underset{C}{\min}\underset{i}{\sum }\frac{1}{2}{\left\|Ub_{i}-Y^{i}c_{i}\right\|}_{2}^{2}+\delta_{1}1_{c}^{T}C1_{*}+\delta_{2}tr\left(CLC^{Τ}\right)+\psi \left(C\right).$$

First, we use the APG method to solve Equation (10) with:

(12) $$F\left(a_{i}\right)=\frac{1}{2}{\left\|Ub_{i}-D^{i}a_{i}\right\|}_{2}^{2}+\lambda_{1}1_{a}^{T}a_{i}+\frac{\lambda_{2}}{2}{\left\|a_{i}-a_{i}^{t-1}\right\|}_{2}^{2}\;G\left(a_{i}\right)=\psi \left(a_{i}\right),$$

where $F\left(a_{i}\right)$ is a differentiable convex function and $G\left(a_{i}\right)$ is a non-smooth convex function. In the APG algorithm, we need to solve an optimization problem:

(13) $$a_{k+1}=\underset{a_{i}}{\mathrm{argmin}}\frac{L}{2}{\left\|a_{i}-\beta_{k+1}+\nabla F\left(\beta_{k+1}\right)/L\right\|}_{2}^{2}+G\left(a_{i}\right),$$

where L (in this paper, L=20) is the Lipschitz constant, k denotes the current iteration time and $\beta_{k+1}$ is defined in Algorithm 1. We define $g_{k+1}=\beta_{k+1}-\nabla F\left(\beta_{k+1}\right)/L$, then the algorithm for solving Equation (7) is given in Algorithm 1.
**Algorithm 1:** Fast numerical algorithm for solving Equation (7).1: **For**
i=1,2,…,N
2: Set $a_{0}=a_{-1}=0\in {\mathbb{R}}^{M}$ and set $\rho_{0}=\rho_{-1}=1$.3: **For**
k = 0,1,…, until converge or a maximal number of iterations have been met4: $\beta_{k+1}=a_{k}+\frac{\rho_{k-1}-1}{\rho_{k}}\left(a_{k}-a_{k-1}\right)$
5: $g_{k+1}=\beta_{k+1}-\frac{1}{L}(D^{i^{T}}\left(D^{i}\beta_{k+1}-Ub_{i}\right)-\lambda_{1}1_{a}-\lambda_{2}\left(\beta_{k+1}-a_{i}^{t-1}\right)$)6: $a_{k+1}=\max\left(0,\;g_{k+1}\right)$
7: $\rho_{k+1}=\left(1+\sqrt{1+4\rho_{k}^{2}}\right)/2$
8: **End**9: Obtain $a_{i}$ via $a_{i}=a_{k+1}$.10: **End**11: Output A


Second, we use the same APG method to solve Equation (11) with:

(14) $$\left(C\right)=\underset{i}{\sum }\frac{1}{2}{\left\|Ub_{i}-Y^{i}c_{i}\right\|}_{2}^{2}+\delta_{1}1_{c}^{T}C1_{c}+\delta_{2}tr\left(CLC^{Τ}\right)\;G\left(C\right)=\psi \left(C\right),$$

Different from Algorithm 1, we need to simultaneously solve all $c_{i}$ in every iteration to exploit the similarity of sparse codes among different layout patches. The key step is to compute the derivative of F(C) versus C. First, we separately compute the derivative of the first term in Equation (14) versus each $c_{i}$:

(15) $$\nabla E\left(c_{i}\right)=Y^{i^{T}}\left(Y^{i}c_{i}-Ub_{i}\right).$$

Then we concatenate all the derivatives to form a derivative matrix $P\left(C\right)=\left[\nabla E\left(c_{1}\right),\nabla E\left(c_{2}\right),\ldots ,\nabla E\left(c_{N}\right)\right]$. The final derivative of F(C) is given as:

(16) $$\nabla F\left(C\right)=P\left(C\right)+\delta_{1}1_{c}1_{*}^{T}+\delta_{2}C\left(L^{T}+L\right),$$

The algorithm for solving Equation (8) is given in Algorithm 2.
**Algorithm 2:** Fast numerical algorithm for solving Equation (8).1: Set $a_{0}=a_{-1}=0\in {\mathbb{R}}^{M\times N}$ and set $\rho_{0}=\rho_{-1}=1$.2: **For**
k = 0,1,…, until converge or a maximal number of iterations have been met3: $\beta_{k+1}=a_{k}+\frac{\rho_{k-1}-1}{\rho_{k}}\left(a_{k}-a_{k-1}\right)$
4: $g_{k+1}=\beta_{k+1}-\frac{1}{L}(P\left(\beta_{k+1}\right)+\delta_{1}1_{c}1_{*}^{T}$ + $\delta_{2}\beta_{k+1}\left(L^{T}+L\right))$
5: $a_{k+1}=\max\left(0,\;g_{k+1}\right)$
6: $\rho_{k+1}=\left(1+\sqrt{1+4\rho_{k}^{2}}\right)/2$
7: **End**8: Obtain C via $C=a_{k+1}$.

(**b**) Fix A and C, solve B: if coefficients A and C are given, Equation (6) turns into the following optimization problem:

(17) $$\underset{b_{i}}{\min}\frac{1}{2}{\left\|Y^{i}c_{i}-Ub_{i}\right\|}_{2}^{2}+\frac{1}{2}\gamma {\left\|D^{i}a_{i}-Ub_{i}\right\|}_{2}^{2}+\mu {\left\|b_{i}\right\|}_{1},\;s.t.i=1,2,\ldots ,N.$$

This sub-problem can also be well solved by the APG method [35] with some customized operations. The customized $F\left(b_{i}\right)$ and $G\left(b_{i}\right)$ are defined as:

(18) $$F\left(b_{i}\right)=\frac{1}{2}{\left\|Y^{i}c_{i}-Ub_{i}\right\|}_{2}^{2}+\frac{1}{2}\gamma {\left\|D^{i}a_{i}-Ub_{i}\right\|}_{2}^{2}\;G\left(b_{i}\right)=\mu {\left\|b_{i}\right\|}_{1}.$$

We define the soft-thresholding operator: $S_{\lambda }\left(x\right)=\mathrm{sign}\left(x\right)\max\left(\left|x\right|-\lambda ,\;0\right)$. Then the algorithm for solving the minimization problem (Equation (17)) is given in Algorithm 3.
**Algorithm 3:** Fast numerical algorithm for solving Equation (17).1: **For**
i=1,2,…,N2: Set $a_{0}=a_{-1}=0\in {\mathbb{R}}^{\left(m\times N\right)\times 1}$ and set $\rho_{0}=\rho_{-1}=1$.3: **For**
k = 0,1,…, until converge or a maximal number of iterations have been met4: $\beta_{k+1}=a_{k}+\frac{\rho_{k-1}-1}{\rho_{k}}\left(a_{k}-a_{k-1}\right)$
5: $g_{k+1}=\beta_{k+1}-\frac{1}{L}\left(U^{T}\left(U\beta_{k+1}-Y^{i}c_{i}\right)+\gamma U^{T}\left(U\beta_{k+1}-D^{i}a_{i}\right)\right)$
6: $a_{k+1}=S_{\mu /L}\left(g_{k+1}\right)$
7: $\rho_{k+1}=\left(1+\sqrt{1+4\rho_{k}^{2}}\right)/2$
8: **End**9: Obtain $b_{i}$ via $b_{i}=a_{k+1}$.10: **End**11: Output B


Finally, the optimization problem in Equation (6) can be iteratively solved by the steps (**a**) and (**b**). The iteration operations are terminated when any of the following two conditions have been met: (1) the difference of objective values between two consecutive iterations is smaller than a threshold (i.e., ${\left\|J^{i}-J^{i-1}\right\|}_{2}\leq \varepsilon$, in this paper, ε is chosen as 0.01); and (2) a maximal number Ω (in this work, Ω=5) of iterations has been met. Details are described in Algorithm 4.
**Algorithm 4:** Algorithm for solving Equation (6).**Input:** The template dictionaries $D^{i}$, the candidate sets $Y^{i}$, the PCA basis dictionary U, the Lipschitz constant L, the occlusion rate vector O and the initiation of B.1: **For**
k=1,2,…, until converge or a maximal number Ω (in this work, Ω=5) of iterations have been met2: Fix $B_{k}$, obtain $A_{k}$ and $C_{k}$ using Algorithms 1 and 2, respectively;3: Fix $A_{k}$ and $C_{k}$ , obtain $B_{k+1}$ by Algorithm 3;4: **End**;5: obtain $\hat{A}$, $\hat{B}$, $\hat{C}$ via $\hat{A}=A_{k-1}$, $\hat{B}=B_{k}$, $\hat{C}=C_{k-1}$;6:**Output:**7: Estimated coefficient matrixes $\hat{A},\hat{B}$ and $\hat{C}$.

### 3.2. Object Tracking via the Proposed MSLM

Our tracking method is based on the Bayesian filtering framework. Similar to [19], we use the affine motion model with six parameters to describe the object’s state $x_{t}={\left[l_{x},l_{y},\theta ,s,r,\phi \right]}^{T}$, where $l_{x},l_{y},\theta ,s,r,\phi$ denote x,y translations, rotation angle, scale, aspect ratio, and skew, respectively. In practice, we randomly sample M particles from a diagonalized Gaussian distribution (i.e., $p\left(x_{t}|x_{t-1}\right)=N\left(x_{t};x_{t-1},\sum )\right)$) to generate a candidate state set $X_{t}=\left[x_{t}^{1},x_{t}^{2},\ldots ,x_{t}^{M}\right]$, where the observation with respect to the $i^{\mathrm{th}}$ candidate is denoted as $z_{i}$. We sample a set of overlapped local image patches inside every candidate region with the spatial layout and convert them into vectors with $\ell_{2}$ normalization, forming a set of candidate patch sets $Y^{i}=\left[y_{1}^{i},y_{2}^{i},\ldots ,y_{M}^{i}\right]\in {\mathbb{R}}^{d\times M}$, where i=1,…,N.

We apply the proposed MSLM and its optimization algorithm on all $Y^{i}$, then we obtain the estimated coefficient matrixes $\hat{A},$ $\hat{B}$**,** and $\hat{C}$.

#### 3.2.1. Occlusion Detection

The estimated sparse PCA coefficients corresponding with each patch are divided into several segments, according to the PCA basis that each segment belongs to, i.e., ${\hat{b}}_{i}^{T}=\left[{\hat{b}}_{i}^{\left(1\right)T},{\hat{b}}_{i}^{\left(2\right)T},\ldots ,{\hat{b}}_{i}^{\left(N\right)T}\right]$, where ${\hat{b}}_{i}^{\left(k\right)}\in {\mathbb{R}}^{m\times 1}$ denotes the $k^{th}$ segment of the estimated coefficient vector ${\hat{b}}_{i}$ and its corresponding PCA basis is $U^{i}$. As $U^{i}$ incrementally learns the appearances of the $i^{th}$ patch and contains no information of other patches, it should have good ability to represent the $i^{th}$ patch, i.e., the coefficients of the PCA basis for the corresponding patch should be larger than others. This means the model is able to deal with partial occlusion. When there is no occlusion, the representation of one patch mainly lies in its corresponding PCA basis. However, when occlusion occurs, the appearance change makes the representation of the occluded local patches dense. Thus, we propose an occlusion metric based on these observations. The occlusion rate of the $i^{th}$ patch is obtained by:

(19) $$o_{i}=\frac{\mathrm{sum}\left({\hat{b}}_{i}\right)-\mathrm{sum}\left({\hat{b}}_{i}^{\left(i\right)}\right)}{\mathrm{sum}\left({\hat{b}}_{i}\right)},$$

where sum(x) means summing all element in vector x together and $o_{i}\in \left[0,1\right]$, the larger $o_{i}$ is, the more severe the occlusion is. Then we get the occlusion rate vector $O={\left[o_{1},o_{2},\ldots ,o_{N}\right]}^{T}$.

#### 3.2.2. Alignment-Weighting Average

**Figure 2:**
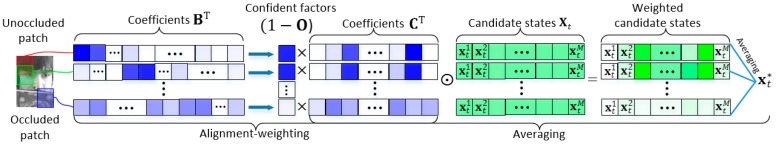
The alignment-weighting average.

Figure 2 shows the flow chart of the alignment-weighting average.

The coefficients in $\hat{C}$ reflect how relevant the corresponding patch is to the target templates and PCA templates. They can be regarded as the confidence scores of these patches belonging to the target object. However, simply summing the coefficients of different patches together as the confidence scores of target candidates is susceptible because if the patch is occluded, the corresponding coefficients are unreliable and, thus, may cause tracking failure. In addition, simply summing the coefficients loses spatial information among different patches. We alleviate these problems by using the occlusion rate of each patch to tune the coefficients. Then we obtain a tuned confidence map $M=\left[m_{1},m_{2},\ldots m_{N}\right]$, where $m_{i}=(1-o_{i})c_{i},\;i=1,2,\ldots ,N$.

Finally, the proposed tracker obtains the optimal state $x_{t}^{*}$ by combining the candidate states with weights based on the tuned confident map, i.e.,:

(20) $$x_{t}^{*}={\left(\varphi \overset{N}{\underset{i=1}{\sum }}\left(m_{i}^{T}X_{t}^{T}\right)\right)}^{T},$$

where φ is a normalized term, equaling to the summation of all elements in M.

#### 3.2.3. Template Update

To account for target appearance variations, we need to update target templates T and PCA basis dictionary U dynamically.

However, the target templates are only obtained from the previous couple of time instants. They can hardly cover the numerous appearance variations of the target object, but they have a strong representation of the current object appearance. Thus, we use them to account for the short-term memory of the target’s appearance. We update T using the method proposed in [30]. This updating strategy can effectively alleviate the influences caused by noise and occlusion.

The PCA Eigen template model has the ability to effectively learning the temporal correlation of target appearances from the past observation data by incremental SVD update procedure. Thus, it can cover a long period of target appearances. We use it to account for the long-term memory of the target. It has been shown [19] that the incremental learning of the PCA subspace representation can effectively and efficiently deal with appearance changes caused by rotations, scale changes, illumination variations, and deformations. In the long-term memory, the new target information used to update the model should be as accurate as possible, because once the wrong information is introduced in the model, it will affect the subsequent tracking results in a long period of time. We label all patches of which their occlusion rates are smaller than θ as positive, and the rest are labelled as negative. In order to obtain precise information, we separately correct each patch with two false rejection operations. First, we identify one patch as false positive when its surrounding patches are all negative ones, then we change its label to negative. Second, we identify one patch as a false negative when its surrounding patches are all positive ones, then we change its label to positive. Finally, we use these collected patches to update their corresponding PCA basis using the method proposed in [19].

## 4. Experiments

**Figure 3:**
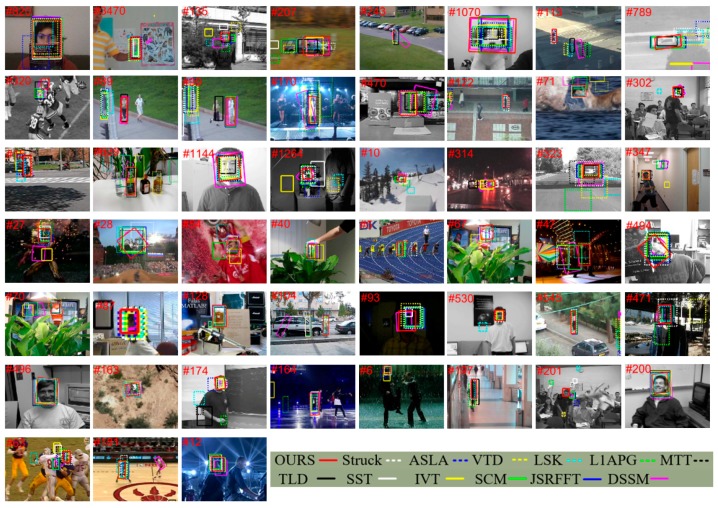
Tracking results of the proposed method and the 12 state-of-the-art tracking methods on representative frames of total 51 sequences in the benchmark [2] (*Football*, *Faceocc1*, *Fish*, *Suv*, *Doll*, *CarScale*, *Jogging-1*, *Subway*, *Jogging-2*, *Crossing*, *Boy*, *Walking*, *Singer1*, *Dog1*, *Deer*, *Freeman3*, *Couple*, *Liquor*, *Mhyang*, *Sylvester*, *Skiing*, *CarDark*, *Car4*, *Boy*, *Ironman*, *MotorRolling*, *Soccer*, *Coke*, *Bolt*, *Tiger1*, *Singer2*, *FaceOcc2*, *Tiger2*, *Girl*, *Lemming*, *David3*, *David*, *David2*, *Woman*, *Trellis*, *Dudek*, *MountainBike*, *Freeman1*, *Skaking1*, *Matrix*, *Walking2*, *Freeman4*, *FleetFace*, *Football1*, *Basketball*, *Shaking*, from left to right, and top to bottom).

The proposed method in this paper is implemented in MATLAB 2014a. We perform the experiments on a PC with Intel i7-4790 CPU (3.6 GHz) and 16 GB RAM memory and the tracker runs at 3.1 fps. We test the performance of the proposed tracker with the total 51 sequences using in the visual tracker benchmark [2] and compare it with the top 12 state-of-the-art trackers, including SST [33], JSRFFT [36], DSSM [27], Struck [9], ASLA [30], L1APG [35], MTT [31], LSK [29], VTD [21], TLD [10], IVT [19], and SCM [37]. Among the 12 selected trackers, the Struck, SCM, TLD, and ASLA are the four best-performed ones demonstrated in the benchmark and our tracker outperforms all of them in terms of the overall performance. Some representative tracking results are shown in Figure 3.

The parameters, which are fixed for each sequence, are summarized as follows. We resize the target image patch to 32×32 pixels and extract 16×16 overlapped local patches within the target region with eight pixels as step length, like in [30]. The number of target templates is set to be 10. The regularization parameters $\lambda_{1}$, $\lambda_{2}$, μ $\delta_{1}$, $\delta_{2}$, and γ are set to be 0.01, 0.01, 0.01, 0.04, 0.2, and 1, respectively. We let the number of PCA basis be 10. The candidate number in each frame is 600. The iteration numbers in Algorithm 1–3 are all set to be 5, and the Lipschitz constant L is equal to 20 for all the three algorithms. Among all the parameters, γ balances the importance between the candidates and the templates. This is a very important factor to our model. We did many experiments to obtain the optimal value of γ. Table 1 summarizes the overall performance of our tracker in terms of γ.

### 4.1. Qualitative Evaluation

The 51 sequences pose many challenging problems, including occlusion (OCC), deformation (DEF), fast motion (FM), illumination variation (IV), scale variation (SV), motion blur (MB), in-plane rotation (IPR), out-of-plane rotation (OPR), background clutter (BC), out-of-view (OV), and low resolution (LR). The distributions of the 51 sequences in terms of the 11 attributes are shown in Table 2.

The most challenging and common problems in tracking are occlusion, deformation, background clutter, illumination change, scale variation, and rotation. We mainly describe how our tracker outperforms the other trackers in these challenging scenarios in details.

**Occlusion:** In 29 of the total 51 sequences, the targets undergo partial or short-term total occlusions. We can see from Figure 3 that the remarkable sparse representation-based trackers (i.e., SCM, DSSM, JSRFFT, SST, ASLA, LSK, L1APG, and MTT) and the well-known incremental subspace-based IVT tracker all fail in some sequences somehow, while our tracker can effectively track almost all of the targets in the 29 sequences when occlusion occurs. This is mainly attributed to the part-based strategy used in our method. The occlusion vector O in Figure 2, which is constructed from the PCA basis coefficients B, can effectively indicate the occlusion degree of each patch. If a patch is occluded, the corresponding element in O will be large, making the tuned confident vector $m_{i}$ very small, thus alleviating the influence of the bad patches. In addition, we exploit the joint-sparsity in patches which are not occluded. This strategy allows the method to fully utilize the spatial information among these patches, making the model more robust.

**Deformation:** There are 19 sequences involve target deformations. We can see from Figure 3 that our tracker can handle deformation better than the other methods. In the *Jogging-1* and *Jogging-2* examples, the proposed method effectively deals with short-term total occlusion when the target undergoes deformation, while most of the other methods fail in these sequences. This is because our method takes advantages of the incremental subspace learning model, which still performs well when deformation occurs.

**Background clutter:** There are total 21 sequences in which the targets suffer background clutter. As the background of the target object becomes complex, it is rather rough to accurately locate the right position of the target, since it is difficult to discriminate the target object from the background in a rather simple model. It is worth noticing that the proposed method performs better than the other algorithms. Thanks to the structural local model and the rich target information preserved in the PCA basis, our model learns a more robust and compact representation of target object, making it easier to capture the target appearance change information.

**Illumination change:** In 25 out of the 51 sequences, the target undergoes severe illumination change. In the *Singer1* sequence our tracker and the IVT tracker performs well in tracking the woman, while many other methods drift to the cluttered background or cannot adapt to scale changes when illumination change occurs. This can be attributed to the use of incremental subspace learning which is able to capture appearance change due to lighting change. In the *Fish* sequence, the target undergoes illumination change together with fast motion. In the *Crossing* sequence, the target has a low resolution observation and goes through illumination change. In all these 25 sequence, our tracker generally outperforms the other trackers.

**Scale variation and rotation:** There are total 44 sequences which undergo scale variation or rotation. As we use the affine transformation parameters that include the scale and rotation sampling, we can capture the candidates with different scales and rotations for further selection. Together with the sampling strategy, the robust representation model proposed in this paper can effectively estimate the current scale and rotation angle of the target object. We also observe that some trackers, including the well-performed Struck tracker, do not adapt to scale or rotation.

### 4.2. Quantitative Evaluation

We use the score of the precision plot and the score of the success plot to estimate the 13 trackers on the 51 sequences. Note that a higher score of the precision plot or a higher score of the success plot means a more accurate result. The overlap rate is defined by $\frac{\mathrm{area}\left(B_{e}\cap B_{g}\right)}{\mathrm{area}\left(B_{e}\cup B_{g}\right)}$, where $B_{e}$ is the estimated bounding box and $B_{g}$ is the ground truth bounding box. We use the precision and success plots used in [2] to demonstrate experiment results of the trackers.

**Figure 4:**
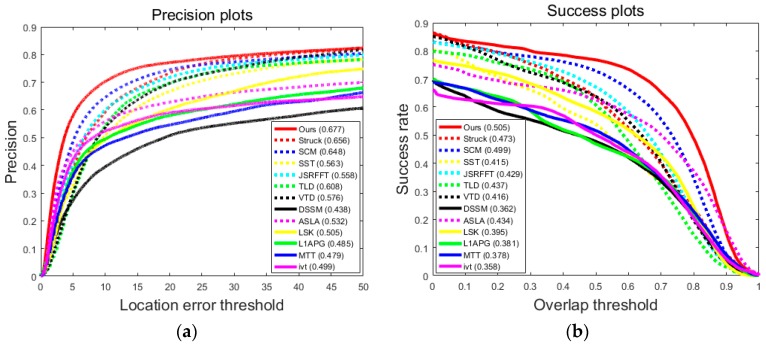
Precision plots (**a**) and success plots (**b**). The legend of the precision plot reports the score of precision plots for each method and the legend of the success plot reports the score of the success plots.

Figure 4 contains the precision plots which show the percentage of frames whose estimated location is within the given threshold distance of the ground truth and success plots which show the ratios of successful frames at the thresholds varied from 0 to 1. Both precision plots and success plots show that our tracker is more effective and robust than the 12 state-of-the-art trackers in terms of the total 51 challenging sequences in the benchmark.

Table 3 and Table 4 report the scores of precision plots and the scores of success plots of different tracking methods. In attributes BC, DEF, IV, IPR, and OV, our tracker achieves the highest scores of precision plots, which means that our method is more robust than the other state-of-the-art trackers. In the MB and LR attributes, the scores of the precision plots of the proposed method are not among the best three. This is because, when undergoing motion blur, different spatial patches of one target tend to have similar blur, making the model distinguish different spatial patches with difficulty. Additionally, along with motion blur, the targets may also go through fast motion or illumination variation. This makes the model even more difficult to accurately track the targets. However, 0.410 of the precision score is still a relatively good one among all of the trackers. In attributes OCC, DEF, IV, IPR, and OV, the proposed tracker achieves the highest scores of success plots which demonstrates that our approach computes the scale more accurately. In the LR attributes, the score of the success plot of the proposed method is also not among the best three. This is because of the low resolution of the target object. Since our tracker is a patch-based method, when the target undergoes low resolution, the patch features will be extracted from even lower resolution patches, resulting in relatively poor representation of each patch, thus causing drift. In the other attributes, our tracker gains the precision scores and success scores very close to the best ones. The last rows of Table 3 and Table 4 show the overall precision scores and success scores of the thirteen trackers over all of the 51 sequences. Our tracker achieves the best scores in both evaluation metrics, which shows that our tracker outperforms all of the other state-of-the-art trackers.

The last row in Table 4 shows the comparison results about computational loads in terms of fps. Our candidate sampling strategy is based on the sampling strategy in [19] and all the candidate patch are resized to 32×32 pixels which means that all of the candidate features are normalized to a fixed size. Thus, the fps of different sequences are the same as long as the candidate numbers are fixed. Actually, we set the candidate number fixed to be 600, so the fps are almost the same in different sequences (ignore the feature extracting time, because it is trivial compared with the time used for solving the whole model.). This shows that our tracker runs at 3.1 fps. Although it does not reach real-time processing, it outperforms most other sparse representation-based trackers (i.e., SCM, MTT, L1APG, DSSM, JSRFFT, and SST) in terms of both accuracy and speed.

## 5. Conclusions

In this paper, we propose a novel multi-view structural local subspace tracking algorithm based on sparse representation. We approximate the optimal state from three views: (1) the template view; (2) the PCA basis view; and (3) the target candidate view. Then we propose a unified objective function to integrate these three view problems together. The model jointly takes advantages of three sub-models by the unified objective function. It not only exploits the intrinsic relationship among target candidates and their local patches, but also takes advantage of both sparse representation and incremental subspace learning. The optimization problem can be solved well by the customized APG methods together with an iteration manner. Then, we proposed an alignment-weighting average method to obtain the optimal state of the target. Furthermore, an occlusion detection strategy is proposed to accurately update the model. Both qualitative and quantitative evaluations demonstrate that our tracker outperforms the state-of-the-art trackers in a wide range of tracking scenarios.

## Figures and Tables

**Table 1:** Overall performance of our tracker in terms of the value of parameter γ.

	5	2	1.3	1.1	1	0.9	0.8	0.5	0.2
**Success Score**	0.277	0.429	0.485	0.491	**0.505**	0.488	0.442	0.416	0.223
**Precision Score**	0.352	0.536	0.604	0.623	**0.677**	0.610	0.582	0.519	0.295

**Table 2:** The distribution of all of the sequences (the number of sequences which have the corresponding attribute).

	OCC	DEF	FM	IV	SV	MB	IPR	OPR	BC	OV	LR
**Total Number**	29	19	17	25	28	12	31	39	21	6	4

**Table 3:** Average precision scores on different attributes: fast motion (FM), scale variation (SV), occlusion (OCC), background clutter (BC), deformation (DEF), motion blur (MB), illumination variation (IV), low-resolution (LR), in-plane rotation (IPR), out-of-plane rotation (OPR), and out-of-view (OV). The best three results are shown in red, blue, and green fonts.

Attributes	IVT	MTT	L1APG	LSK	ASLA	DSSM	VTD	TLD	JSRFFT	SST	SCM	Struck	OURS
**FM**	0.220	0.413	0.365	0.375	0.253	0.397	0.353	0.551	0.401	0.393	0.331	0.604	0.439
**SV**	0.494	0.461	0.472	0.480	0.552	0.422	0.597	0.606	0.513	0.541	0.672	0.639	0.647
**OCC**	0.455	0.433	0.461	0.534	0.460	0.401	0.546	0.563	0.557	0.486	0.639	0.565	0.572
**BC**	0.421	0.424	0.425	0.504	0.496	0.319	0.571	0.428	0.511	0.503	0.578	0.585	0.591
**DEF**	0.409	0.332	0.383	0.481	0.445	0.519	0.501	0.512	0.482	0.521	0.586	0.521	0.597
**MB**	0.222	0.308	0.375	0.324	0.278	0.320	0.375	0.518	0.440	0.426	0.339	0.551	0.410
**IV**	0.418	0.359	0.341	0.449	0.516	0.359	0.557	0.537	0.307	0.560	0.592	0.558	0.606
**LR**	0.278	0.510	0.460	0.304	0.156	0.358	0.168	0.349	0.546	0.274	0.305	0.545	0.385
**IPR**	0.457	0.528	0.518	0.534	0.511	0.405	0.600	0.584	0.510	0.584	0.596	0.617	0.621
**OPR**	0.464	0.478	0.478	0.525	0.518	0.319	0.620	0.596	0.493	0.532	0.617	0.597	0.599
**OV**	0.307	0.374	0.329	0.515	0.333	0.384	0.462	0.576	0.396	0.490	0.429	0.539	0.582
**Overall**	0.499	0.479	0.485	0.505	0.532	0.438	0.576	0.608	0.558	0.563	0.648	0.656	0.677

**Table 4:** Average success scores on different attributes: fast motion (FM), scale variation (SV), occlusion (OCC), background clutter (BC), deformation (DEF), motion blur (MB), illumination variation (IV), low-resolution (LR), in-plane rotation (IPR), out-of-plane rotation (OPR), out-of-view(OV). The best three results are shown in red, blue, and green fonts. The last row shows comparison results regarding computational loads in terms of fps.

Attributes	IVT	MTT	L1APG	LSK	ASLA	DSSM	VTD	TLD	JSRFFT	SST	SCM	Struck	OURS
**FM**	0.202	0.338	0.311	0.328	0.248	0.332	0.303	0.420	0.341	0.343	0.296	0.461	0.428
**SV**	0.344	0.348	0.350	0.373	0.452	0.318	0.405	0.424	0.367	0.405	0.518	0.425	0.427
**OCC**	0.325	0.345	0.353	0.409	0.376	0.349	0.404	0.405	0.411	0.365	0.487	0.412	0.492
**BC**	0.291	0.337	0.350	0.388	0.408	0.321	0.425	0.348	0.401	0.394	0.450	0.458	0.435
**DEF**	0.281	0.280	0.311	0.377	0.372	0.342	0.377	0.381	0.360	0.382	0.448	0.393	0.451
**MB**	0.197	0.274	0.310	0.302	0.258	0.297	0.309	0.407	0.313	0.336	0.298	0.433	0.397
**IV**	0.306	0.308	0.283	0.371	0.429	0.317	0.420	0.402	0.291	0.437	0.472	0.427	0.489
**LR**	0.238	0.389	0.381	0.235	0.157	0.284	0.177	0.312	0.392	0.191	0.279	0.372	0.370
**IPR**	0.330	0.398	0.391	0.411	0.425	0.347	0.430	0.419	0.447	0.413	0.457	0.443	0.458
**OPR**	0.323	0.364	0.360	0.400	0.422	0.331	0.435	0.423	0.411	0.409	0.470	0.431	0.436
**OV**	0.274	0.342	0.303	0.430	0.312	0.348	0.446	0.460	0.350	0.384	0.361	0.459	0.463
**Overall**	0.358	0.378	0.381	0.395	0.434	0.362	0.416	0.437	0.429	0.415	0.499	0.473	0.505
**FPS**	30.9	1.2	2.1	5.3	8.8	1.2	5.8	29.1	1.7	1.3	0.6	22.4	3.1
